# Thrombo-Inflammatory Extracellular Vesicles 2.0

**DOI:** 10.3390/ijms242216134

**Published:** 2023-11-09

**Authors:** Tommaso Neri

**Affiliations:** Centro Dipartimentale di Biologia Cellulare Cardiorespiratoria, Dipartimento di Patologia Chirurgica, Medica, Molecolare e dell’Area Critica, Università degli Studi di Pisa, 56124 Pisa, Italy; tommaso.neri@unipi.it

In the complex field of cell-to-cell communication and physiological regulation, there is a remarkable category of tiny messengers called extracellular vesicles (EVs) [1]. These microscope structures, secreted by virtually all cell types, carry a cargo of biological molecules, including proteins, lipids, and genetic material [2]. EVs serve as couriers, distributing essential information and regulating a diverse range of physiological activities. One of the most intriguing areas where EVs have recently emerged as key players is thromboinflammation [3]. Thromboinflammation refers to the intricate interplay between blood clot formation (thrombosis) and inflammation, two fundamental defense mechanisms in the human body. While both processes are vital for protecting against infections and injuries, their dysregulation can lead to severe health issues, such as sepsis, cardiovascular diseases, and even complications in diseases like COVID-19 [4]. EVs are closely involved in the orchestration of thromboinflammation. They serve as critical intermediaries, shuttling bioactive molecules that affect platelet activation, immune response, and the formation of blood clots. By collaborating in this delicate balance between coagulation and inflammation, EVs are promising candidates for comprehension and possibly controlling thromboinflammation. 

Recent developments in this field have opened new horizons for understanding the role of EVs in the complex dance of thromboinflammation. 

Despite the strides made, our comprehension of thromboinflammation and extracellular vesicles remains incomplete. Key gaps in our knowledge persist, including the identification of specific EV markers associated with thromboinflammation, the potential for EVs as therapeutic targets is promising, but further research is needed to translate these findings into clinical applications effectively and finally understand the functional significance of EVs in different thromboinflammatory conditions is crucial.

In this Special Issue, we begin an investigation of EVs and their pivotal role in guiding the intricate dance between thrombosis and inflammation. 

The Special Issue includes a total of six contributions, each with its unique focus and insights, collectively illuminate the multifaceted nature of EVs in the context of thromboinflammation: three original articles and three reviews. Collectively, these studies expand our knowledge of the intricate world of EVs and their multifaceted roles in various health conditions, from cardiovascular diseases to respiratory disorders, and even in the context of infectious diseases like COVID-19. They highlight the importance of continued research into EVs, offering a promising avenue for potential therapeutic interventions and a deeper understanding of complex medical conditions. 

In conclusion, the nexus of thromboinflammation and extracellular vesicles is a dynamic field brimming with potential. This Special Issue can offer a compass for navigating this complex terrain, emphasizing the need for continued research to fill the existing knowledge gaps. As we look to the future, these insights promise to guide the development of more effective diagnostics and treatments, ultimately improving the quality of healthcare and patient outcomes.
